# Investigating the association between physicians self-efficacy regarding communication skills and risk of “burnout”

**DOI:** 10.1186/s12955-020-01504-y

**Published:** 2020-08-06

**Authors:** Andrea Messerotti, Federico Banchelli, Silvia Ferrari, Emiliano Barbieri, Francesca Bettelli, Elena Bandieri, Davide Giusti, Hillary Catellani, Eleonora Borelli, Elisabetta Colaci, Valeria Pioli, Monica Morselli, Fabio Forghieri, Gian Maria Galeazzi, Roberto Marasca, Sarah Bigi, Roberto D’Amico, Peter Martin, Fabio Efficace, Mario Luppi, Leonardo Potenza

**Affiliations:** 1 Hematology Unit and Chair, Azienda Ospedaliera Universitaria di Modena and Department of Medical and Surgical Sciences, University of Modena and Reggio Emilia, Modena, Italy; 2 Department of Medical and Surgical Sciencesm, Statistic Unit, University of Modena and Reggio Emilia, Modena, Italy; 3 Department of Biomedic and Metabolic Science and Neuroscience, section of Psychiatry, University of Modena and Reggio Emilia, Modena, Italy; 4 Oncology and Palliative Care Units, Civil Hospital Carpi, Local Health Agency, Carpi (Modena), Italy; 5 Department of Biomedic and Metabolic Science and Neuroscience, University of Modena and Reggio Emilia, Modena, Italy; 6 Department of Linguistic Sciences and Foreign Literatures, Catholic University of the Sacred Heart, Milan, Italy; 7 Centre for Organisational Change in Person-Centred Healthcare, Faculty of Health, Deakin University, Melbourne, Australia; 8 Italian Group for Adult Hematologic Disease (GIMEMA), Health Outcomes Research Unit, Rome, Italy

**Keywords:** Breaking serious news, Attitudes, Burnout, Communication skills

## Abstract

**Background:**

Breaking bad news (BBN) may be associated with increasing risk of burnout in practising physicians. However, there is little research on the association between the way bad news is broken and burnout. We investigated the association between physicians’ self-efficacy regarding communication to patients and risk of burnout.

**Methods:**

We performed a cross-sectional study by proposing an ad-hoc survey exploring attitudes and practice regarding BBN and the Maslach Burnout Inventory - Human Service Survey to 379 physicians from two University Hospitals in Italy. Associations were assessed by multivariable logistic regression models.

**Results:**

Two-hundred twenty-six (60%) physicians returned the questionnaires. 76% of physicians acquired communication skills by observing mentors or colleagues, 64% considered BBN as discussing a poor prognosis, 56% reported discussing prognosis as the most difficult task, 38 and 37% did not plan a BBN encounter and considered it stressful. The overall burnout rate was 59%. Considering BBN a stressful task was independently associated with high risk of burnout (OR 3.01; *p* = 0.013). Planning the encounter (OR = 0.43, *p* = 0.037), mastering communication skills (OR = 0.19, *p* = 0.034) and the self-evaluation as good or very good at BBN (OR 0.32; 0.15 to 0.71; *p* = 0.0) were associated with low risk of burnout.

**Conclusions:**

Our findings suggest that some physicians’ BBN attitudes and knowledge of conceptual frameworks may influence the risk of burnout and support the notion that increasing knowledge about communication skills may protect clinicians from burnout. Further research is needed in this area.

## Background

Burnout is a psychological work-related syndrome typically affecting the helping professions, characterised by 3 core dimensions: physical and emotional exhaustion (PEE), cynicism and depersonalization (CD), and low personal accomplishment (PA) [1, 2]. Recent studies have reported that more than 50% of medical doctors suffer from burnout. Such an epidemic negatively affects patient care, professionalism, physicians’ health and safety, and the viability of health-care systems. Numerous individual and work-related factors contribute to develop the burnout of clinicians [3]. One of the most frequently advocated stressor is breaking bad news (BBN) [2, 3].

BBN, such as discussing diagnosis, disclosing a poor prognosis or discussing the transition to palliative care with patients and their families, is a core communication task in medicine [4]. The ability of physicians to deliver bad news has been studied with surveys exploring mainly their self-efficacy, intended as the beliefs in their capacity to execute such a task and their expectation of being able to successfully perform that behaviour according to experiences and/or training [5–7]. The way BBN is conveyed may seriously affect patients and families [8]. However, BBN have consequences also for physicians, who may experience strong emotions and distress. By harnessing simulation methodologies and measuring physiological indices, such as heart rate and sweating indices, several studies have empirically demonstrated that BBN may provoke fear, anxiety, discomfort and burden of responsibility in physicians. All these causes of distress may ultimately lead to burnout, with detrimental consequences on clinical effectiveness [9–11]. Nonetheless, the association between the way serious news are broken and burnout has not yet been explored [12].

We have sought to examine the association between the frameworks and professional development opportunities physicians utilise regarding healthcare communication (HC) and how that relates to a metric linked with burnout.

## Methods

### Characteristics of the study

The study is a cross-sectional survey study enrolling physicians working in two tertiary care hospitals (AOU-Policlinico di Modena and AOU-Ospedale Civile di Baggiovara) in Modena, Italy. The study was approved by the local Ethical Committee (CE protocol n° 244/16). An informed consent was obtained from physicians participating in the study. Participation was voluntary, anonymous and no incentive was offered. The survey was delivered by e-mail or directly to the ward, according to the number of physicians we knew working in that specific ward, and it was returned with the same procedures.

### Study population

**Fig. 1:**
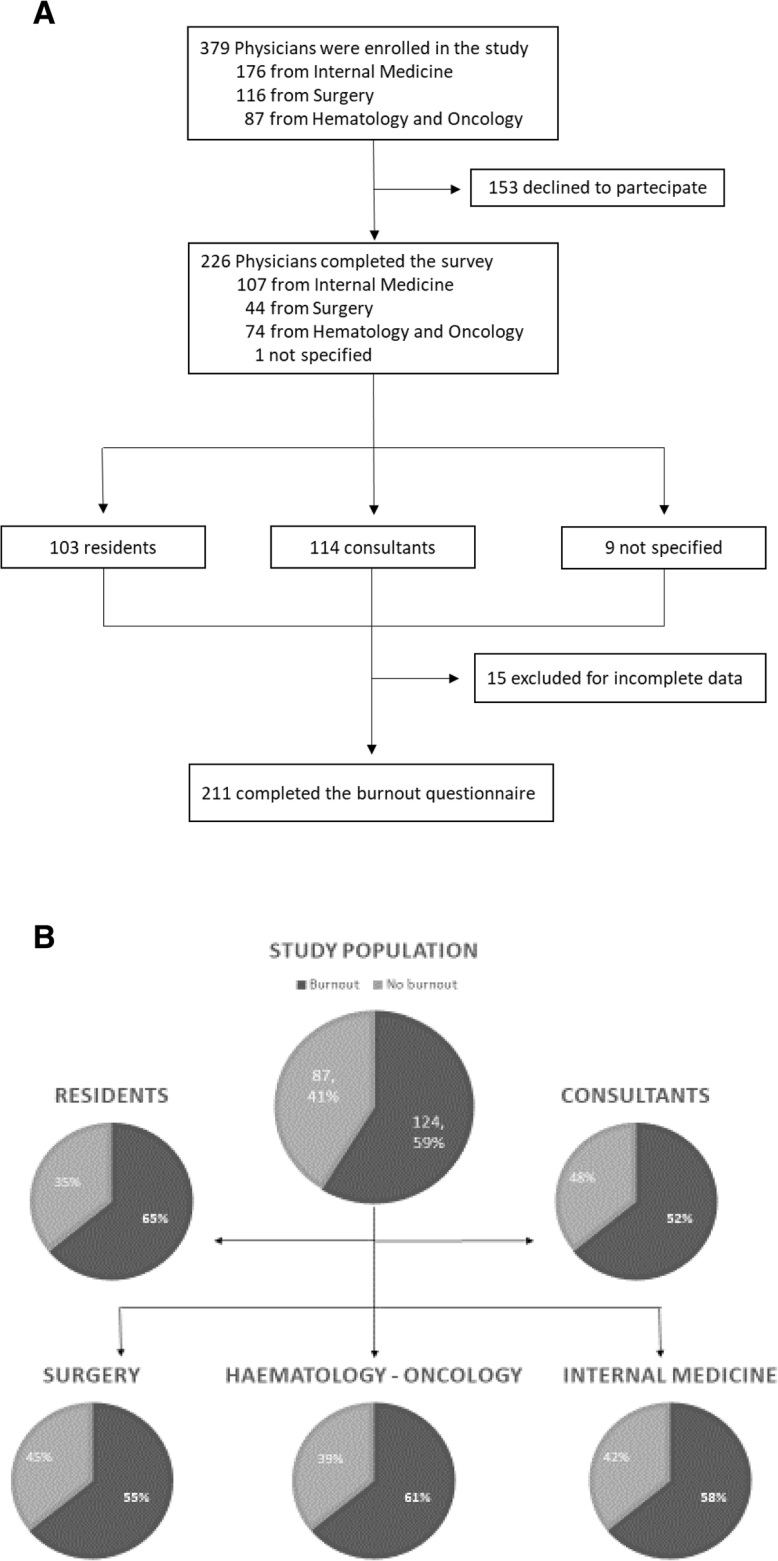
**a,b**. Study design flowchart and burnout analysis. **a**. The flowchart shows physicians’ characteristics and responses at the questionnaires. **b**. Burnout rates in consultants and residents and according to their branch of medical practice. Dark grey segments represent physicians with burnout in at least one dominion. Light grey segments represent physicians without burnout

Three-hundreds-seventy-nine physicians were enrolled into the study. Of them, 226 (60%) completed the survey (Fig. 1a). A complete description of the sample is provided in Table 1. As the survey was anonymous, we were unable to evaluate the characteristics of physicians who did not return the questionnaire. The clustering of the years from graduation into 4 levels was aimed to group together physicians with supposed similar levels expertise in and development opportunities and acquisition of communication skills.

**Table 1:** Characteristics of Physicians

	(N/%)
Physicians enrolled	379
Physicians returning the questionnaire	226(60)
**Specialty**	
Internal Medicine area	107(47)
Haematology/Oncology area	74(33)
Surgical area	44(20)
Unspecified	1
**Gender**	
Male	100(44)
Female	116(51)
Unknown	**10(5)**
**Professional Role**	
Resident	103(46)
Consultant	114(50)
Not specified	9(4)
**Years from graduation**	
≤ 3	55(24)
> 3 and ≤ 6	45(20)
> 6 and ≤ 16	60(26)
> 16	62(28)
Not specified	4(2)

Internal medicine area includes Internal Medicine, Pneumology, Infectivology, Emergency Medicine, Nefrology, Gastroenterology and Endocrinology; Haematology/Oncology area includes Haematology and Oncology; Surgical Area includes Otorhinolaryngology, Plastic Surgery, General Surgery, Thoracic Surgery and Orthopaedics

### Survey instruments

#### Clinician-perceived communication skills questionnaire

No validated instruments for measuring clinicians’ communication skills have been developed. An ad-hoc survey was developed by the authors, including a psychiatrist specialized in burnout and assisting cancer and terminal-ill patients and physicians specialized in providing specific supportive care with an advanced training in communication skills. In particular, one of them underwent a faculty development course of VitalTalk (www.vitaltalk.org) and all the other underwent communication skills training according to the same model. The Oncotalk/VitalTalk teaching model is based on evidence-based principles and includes brief didactic sessions to provide specific communication skills, demonstration of those skills by faculties, intensive skill practice with simulated patients during which group and faculties give feedbacks to the trainee focusing on trainee’s needs and attending to trainee’s attitudes and emotions [13]. The survey was based on previously published researches exploring physician communication of bad news through self-administered questionnaires, investigating the attitudes and problems in disclosure BBN, perceived confidence and outcome of physicians’ own communication skills, knowledge and self-efficacy about BBN, the usual practice, frequencies and format of communication with patients and/or family members [4, 14–20].

The tool is composed by a 23-item questionnaire for assessing physicians’ perceptions of their communication skills (CS) knowledge and self-rating of HC. The questionnaire was strictly confidential and anonymous. The following steps and key aspects of a clinician-patient encounter were investigated: 1) plan the encounter, 2) BBN, 3) discussing prognosis, 4) shared decision making process, 5) tracking and responding to emotions, 6) communication skill training (CST), 7) self-evaluation about communication skills. 16 out of 23 items allowed multiple answers and 7 had only one possible answer.

#### Burnout questionnaire

Burnout was measured using the validated Italian version of the Maslach Burnout Inventory - Human Services Survey (MBI-HSS), 22-items [1, 21]. The standard scoring for health care workers was used. Burnout syndrome was considered present if at least one of the three dimensions was severely abnormal, according to criteria proposed by Grunfeld et al. [21].

### Statistical analysis

Descriptive statistics of the study sample were calculated; mean and standard deviation were used for continuous variables, whereas absolute and percentage frequencies were used for categorical variables. Results were expressed in terms of odds ratios (OR) with 95% confidence interval (95% CI) and associated *p*-values, comparing each modality with the reference modality. Association between our observed covariates and the presence of burnout was assessed by means of logistic regression models. First, a single-item analysis was performed, where the dependent variable was a positive score for burnout and the independent variables were the items of the communication skills questionnaire. The single-item analysis was carried out for all the 23 items of the questionnaire. Finally, a multivariable analysis was also performed, by considering a positive score for burnout as the dependent variable, while 8 items of the communication skills questionnaire as well as being a resident or a consultant as the independent variables. The 8 items, for a total of 25 covariates, were the following: 1, 4, 7, 9, 16, 19, 20, 22. These items were chosen because they resulted statistically significant in the single-item analysis. Only 2 items associated with measures of burnout in the single-item analysis, namely n 11 and n 23, were excluded, due to high rate of missing data, to maintain the ratio between subjects with burnout/evaluated covariates greater than five and to avoid the risk of multicollinearity in the covariates. Goodness-of-fit of our multivariable model was measured by means of the c-statistic (i.e. area under the ROC curve). Data were analysed by means of the R 3.4.3 software (The R Foundation for Statistical Computing, Wien).

## Results

### Communication skills questionnaire

A full report of the results is included in Table 2.

**Table 2:** Physicians’ communication preferences

	IMA^a^	HOA^b^	SA^c^	TOT	IMA %	HOA%	SA %	TOT %
**Planning the encounter**								
**1**. How do you prepare for breaking bad news encounters?								
Have a consistent plan or strategy	40	32	14	**86**	37%	43%	32%	**38%**
No consistent approach to task	42	30	15	**87**	39%	41%	34%	**39%**
Use my experience	19	5	3	**27**	18%	7%	7%	**12%**
Follow my emotions	7	6	7	**20**	7%	8%	16%	**9%**
Plan to provide all relevant information at once then respond to questions	22	17	13	**52**	21%	23%	30%	**23%**
**2**. In your opinion, would a strategy or approach to breaking bad news be important?								
Yes	67	47	25	**139**	63%	64%	57%	**62%**
No	10	1	4	**15**	9%	1%	9%	**7%**
Maybe	28	20	13	**61**	26%	27%	30%	**27%**
Don’t know	2	6	2	**10**	2%	8%	5%	**4%**
**3**. In your opinion, why physicians do not use a strategy or approach to breaking bad news?								
Lack of time	32	27	17	**76**	30%	36%	39%	**34%**
Not necessary	26	22	10	**58**	24%	30%	23%	**26%**
Can’t say	20	13	9	**42**	19%	18%	20%	**19%**
Not to put distance between themselves and the patient	23	15	7	**45**	21%	20%	16%	**20%**
Don’t consider breaking bad news a clinical skill	19	9	4	**32**	18%	12%	9%	**14%**
**Breaking bad news**								
**4**. What does breaking bad news mean for you?								
Discussing diagnosis	22	17	5	**44**	21%	23%	11%	**20%**
Telling patient he/she is terminally ill	45	29	11	**85**	42%	39%	25%	**38%**
Discussing a poor prognosis	71	47	27	**145**	66%	64%	61%	**64%**
Talking about end of active treatment	57	47	16	**120**	53%	64%	36%	**53%**
Discussing diagnosis of cancer	35	25	5	**65**	33%	34%	11%	**29%**
**5**. In an average month, how often do you have to break bad news to a patient/family?								
Never	6	0	10	**16**	6%	0%	23%	**7%**
1 to 5 times	69	33	21	**123**	64%	45%	48%	**55%**
5 to 10 times	26	21	7	**54**	24%	28%	16%	**24%**
More than 10 times	6	20	6	**32**	6%	27%	14%	**14%**
**6**. Which one do you think is the most difficult task of breaking bad news?								
Discussing prognosis	60	45	20	**125**	56%	61%	45%	**56%**
Telling patient about recurrence	19	26	15	**60**	18%	35%	34%	**27%**
Discussing transition to palliative care	30	42	15	**87**	28%	57%	34%	**39%**
Encouraging and dealing with family involvement	15	10	5	**30**	14%	14%	11%	**13%**
Discussing diagnosis	22	11	6	**39**	21%	15%	14%	**17%**
**7**. How would you describe the part of your job in which you break bad news?								
Stimulating	4	5	2	**11**	4%	7%	5%	**5%**
Stressful	36	33	14	**83**	34%	45%	32%	**37%**
Emotionally engaging	78	60	30	**168**	73%	81%	68%	**75%**
Worrisome	6	4	2	**12**	6%	5%	5%	**5%**
Depressing	9	5	3	**17**	8%	7%	7%	**8%**
**8**. What do you feel is the most difficult part of breaking bad news?								
Being honest but not taking away hope	75	55	32	**162**	70%	74%	73%	**72%**
Dealing with the patient’s emotions	27	26	6	**59**	25%	35%	14%	**26%**
Spending the right amount of time	8	15	9	**32**	7%	20%	20%	**14%**
Involving friends and family of the patient	3	3	0	**6**	3%	4%	0%	**3%**
Involving patient or family in decision making	13	5	5	**23**	12%	7%	11%	**10%**
**Discussing prognosis**								
**9**. What does discussing prognosis mean for you?								
Information about illness trajectory and outcome	56	42	19	**117**	52%	57%	43%	**52%**
Success/failure rates of treatment options	61	49	24	**134**	57%	66%	55%	**60%**
Mean survival time for patients affected by the same disease and undergoing the same treatment	20	14	6	**40**	19%	19%	14%	**18%**
Chances of cure	27	13	5	**45**	25%	18%	11%	**20%**
Success rates of treatment options	37	32	11	**80**	35%	43%	25%	**36%**
**10**. Would you inform patient and family about prognosis?								
Yes, certainly	67	44	28	**139**	63%	59%	64%	**62%**
No	3	1	2	**6**	3%	1%	5%	**3%**
Patient no, family yes	13	11	5	**29**	12%	15%	11%	**13%**
Family no, patient yes	5	1	2	**8**	5%	1%	5%	**4%**
Only if patient/family asks about it	12	20	5	**37**	11%	27%	11%	**16%**
Only under certain circumstances	3	1	1	**5**	3%	1%	2%	**2%**
**11**. If yes, for which reason?								
Ethical reasons	14	10	6	**30**	13%	14%	14%	**13%**
Foster therapeutic compliance	21	13	6	**40**	20%	18%	14%	**18%**
Improve patient’s awareness of treatment plan	23	22	17	**62**	21%	30%	39%	**28%**
Make patient aware of illness trajectory, therapeutic choices and optimize adjustment to new conditions	65	42	20	**127**	61%	57%	45%	**56%**
**12**. If not, for which reason?								
Physicians are not updated about diseases prognosis	2	0	0	**2**	3%	0%	0%	**1%**
Physicians do not know how to discuss prognosis	1	2	1	**4**	2%	3%	5%	**3%**
Lack of time	0	1	0	**1**	0%	2%	0%	**1%**
Not to take away hope	10	13	5	**28**	16%	22%	24%	**20%**
Not to scare patients	4	7	0	**11**	7%	12%	0%	**8%**
Patients might not be ready	12	13	6	**31**	20%	22%	29%	**22%**
Patients might not be able to handle emotions	16	13	6	**35**	26%	22%	29%	**25%**
Physicians cannot know every single patient’s prognosis	12	5	1	**18**	20%	8%	5%	**13%**
Physicians do not ask how patients want to discuss prognosis	5	5	2	**12**	8%	8%	10%	**9%**
**Sharing decision making**								
**13**. Do you usually ask patients how much they want to know before breaking bad news?								
Yes	23	25	11	**59**	21%	33%	25%	**26%**
No	84	50	33	**167**	79%	67%	75%	**74%**
**14**. In your opinion, why do not physicians ask patients how much they want to know?								
They can understand it all by themselves	23	19	5	**47**	21%	26%	11%	**21%**
Physicians always tell what they consider necessary	30	27	16	**73**	28%	36%	36%	**32%**
Patients might get scared by that question	45	27	12	**84**	42%	36%	27%	**37%**
Patients are always informed by physicians	28	17	19	**64**	26%	23%	43%	**28%**
**15**. In an average month, how often do you talk to patients who do not want to receive information about their disease?								
Less than 5 times	100	54	42	**196**	93%	74%	95%	**87%**
5 to 10 times	4	17	1	**22**	4%	23%	2%	**10%**
10 to 20 times	2	1	1	**4**	2%	1%	2%	**2%**
More than 20 times	1	1	0	**2**	1%	1%	0%	**1%**
**16**. What do you offer when discussing treatment options?								
The best treatment for the patient, to the best of my knowledge and belief	72	52	33	**157**	67%	70%	75%	**70%**
To choose between all the available treatment options	17	5	8	**30**	16%	7%	18%	**13%**
To share decision with me	45	36	8	**89**	42%	49%	18%	**40%**
To trust my opinion	0	0	1	**1**	0%	0%	2%	**0%**
The most innovative treatment option	1	0	0	**1**	1%	0%	0%	**0%**
**17**. At the end of a visit, how often do you check for patient understanding?								
Every time	43	22	16	**81**	40%	30%	36%	**36%**
Never	2	1	1	**4**	2%	1%	2%	**2%**
Every time I think patient is not understanding	61	50	24	**135**	57%	68%	55%	**60%**
Every time I notice patient has limited health literacy	12	5	2	**19**	11%	7%	5%	**8%**
When patient asks me weird questions	13	9	4	**26**	12%	12%	9%	**12%**
**Tracking and responding to emotions**								
**18**. Which of the following emotions do patients show you more often?								
Fear	76	58	30	**164**	71%	78%	68%	**73%**
Anger	14	24	3	**41**	13%	32%	7%	**18%**
Sadness	34	31	16	**81**	32%	42%	36%	**36%**
Disgust	0	1	1	**2**	0%	1%	2%	**1%**
Happiness	6	5	0	**11**	6%	7%	0%	**5%**
Disappointment	13	15	5	**33**	12%	20%	11%	**15%**
**19**. What do you do when patients show you their feelings?								
Talk about the benefits of therapy	12	6	8	**26**	11%	8%	18%	**12%**
Remain silent waiting for the end	15	15	4	**34**	14%	20%	9%	**15%**
Address patients’ emotions with empathic responses	74	49	22	**145**	69%	66%	50%	**64%**
Highlight what is positive	41	27	18	**86**	38%	36%	41%	**38%**
Interrupt the visit then start again when patients are more relaxed	1	1	0	**2**	1%	1%	0%	**1%**
**Communication skills training**								
**20**. How did you develop your communication skills?								
Observing mentors and older colleagues	78	61	31	**170**	73%	82%	70%	**76%**
Experience	58	41	17	**116**	54%	55%	39%	**52%**
Communication skills training courses	8	7	0	**15**	7%	9%	0%	**7%**
Textbooks and scientific literature	6	4	4	**14**	6%	5%	9%	**6%**
Medical school	3	6	5	**14**	3%	8%	11%	**6%**
**21**. Would a strategy or approach to breaking serious news be helpful in your practice?								
Yes, certainly	76	60	34	**170**	70%	81%	77%	**75%**
No	3	0	0	**3**	3%	0%	0%	**1%**
It is not possible to determine in advance a way to do it regardless of the situation and the individual needs.	29	14	10	**53**	27%	19%	23%	**24%**
**Self-evaluation**								
**22**. How do you feel about your own ability to break serious news?								
Very good	2	0	2	**4**	2%	0%	5%	**2%**
Good	32	26	16	**74**	30%	35%	36%	**33%**
Fair	57	32	21	**110**	53%	43%	48%	**49%**
Poor	8	9	2	**19**	7%	12%	5%	**8%**
Very poor	9	7	3	**19**	8%	9%	7%	**8%**
**23**. In a qualitative study on patient-physician relationship, patients have been asked to “classify” their physicians basing on the attitudes and skills physicians showed them during treatments.^[26]^ Which kind of physicians do you think you are?								
Unskilled	25	14	6	**45**	24%	21%	14%	**21%**
Emotionally overwhelmed	4	2	1	**7**	4%	3%	2%	**3%**
Tough but skillful	6	4	6	**16**	6%	6%	14%	**8%**
Insensitive but skillful	4	1	2	**7**	4%	1%	5%	**3%**
Detached	6	1	1	**8**	6%	1%	2%	**4%**
Empathic and professional	59	45	26	**130**	57%	67%	62%	**61%**

*Abbreviations*: *IMA* Internal Medicine Area, *HOA* Haematology/Oncology Area, *SA* Surgical Area

Among the most notable answers, there were the following: in the “plan the encounter” section, 139 physicians (62%) considered important to have a plan before BBN encounter. However, only 86 (38%) admitted preparing one, while 87 (39%) reported not to have a plan for the encounter, providing lack of time (*N* = 76, 34%) and the idea that planning may not be necessary (*N* = 58, 26%) as the main causes.

When asked about “definition of BBN”, 145 (64%) and 120 (53%) physicians answered that BBN means discussing a poor prognosis or talking about the end of disease-modifying treatment, respectively. Discussing prognosis and transition to palliative care were considered to be the most difficult tasks of BBN by 125 (56%) and 87 (39%) physicians. 168 (75%) of interviewees described BBN as emotionally engaging and 83 (37%) stressful. The most difficult part of BBN was balancing hope with honesty for 162 (75%) physician. 59 (26%) reported this was dealing with patients’ emotions.

As to “discussing prognosis”, 139 (62%) physicians would be in favour of informing both patients and families about prognosis, mainly because they believe it promotes patients’ coping skills and empowerment. Nevertheless, 125 (56%) physicians acknowledged that they disclose prognosis only by talking about the rates for cure and response of treatment options.

When asked about “sharing decision making”, 167 physicians (74%) revealed they do not usually ask patients how much information they want to know before BBN, mainly because they think that it is already felt by patients as worrisome, and patients may get scared simply by such question [84 (37%)]. As to discussing treatment options, 157 (70%) physicians just recommended the best treatment, in their opinion for the patients, while 89 (40%) attempted to share the decision making. Only 81 (36%) declared to check the patients understanding at the end of every visit.

Regarding to “tracking and responding to emotions”, 164 (73%) physicians thought fear to be the most common emotion showed by patients. Overall, 145 (64%) reported to address patients’ emotions with empathic responses.

The vast majority of respondent (170, 76%) based their HC professional development by observing colleagues and/or relied on experience. Only 15 (7%) and 14 (6%) physicians, respectively, reported attended CS training courses or receiving this training in Medical Schools. 14 (6%) relied on learning CS from textbooks or the scientific literature.

188 (84%) physicians considered themselves to be at least fair at BBN and 130 (61%) to be empathic and professional, while 45 (21%) acknowledged themselves to be unskilled for the task. Three quarters of the sample admitted not having an evidence based approach and that a strategy to BBN would be helpful in their clinical practice.

### Burnout

211 (93%) out of 226 questionnaires were fully evaluable for analysis. 124 (59%), physicians reached clinical significance of burnout in at least one of the 3 dimensions. In details, 66 (53%) out of 124 in 1 dimension; 46 (37%) in 2 dimensions and 12 (10%) in all 3 dimensions. Burnout levels of junior doctor, while they are acquiring specialization, were higher than those of consultants in a statistically significant manner (60 (65%) out of 99 vs 57 (52%) out of 109; OR 1.75; 95% CI 1.00 to 3.04; *p* = 0.049) (Fig. 1b).

### Associations between physicians’ communication skills and burnout

#### Single-item analysis

In our single-item analysis, the following variables were related to high levels of burnout: 1) physicians believing that BBN means discussing a poor prognosis (*p* = 0.039); 2) physicians self-assessing BBN to be a stressful task for themselves (*p* = 0.001); 3) discussing prognosis only including the rates for cure (*p* = 0.036); 4) feeling unskilled at patient-physician relationship (*p* = 0.029) and 5) being a resident (*p* = 0.049). On the contrary, the following variables were found to be related to low levels of burnout: 1) considering BBN an emotionally engaging task (*p* = 0.042); 2) having a consistent plan for communicating with patients (*p* = 0.040); 3) responding to patients’ emotions with empathic responses (*p* = 0.017); 4) discussing prognosis with the goal of promoting awareness of illness trajectory, therapeutic choices and to optimize patients’ coping (*p* = 0.010); 5) sharing decisions with patients (*p* = 0.019); 6) developing CS by using textbooks and scientific literature (*p* = 0.011); 7) feeling to be good or very good at CS (*p* = 0.000); 8) graduation within the last 6 to 16 years (*p* = 0.003) (Table 3).

**Table 3:** Associations between communication patterns and burnout: single-item analysis

Variables	All physicians (***n*** = 211) N (%)	Physicians with burnout (***n*** = 124) N (%)	OR	95% CI	***P*** value
**Factors associated with high risk of burnout**					
Breaking bad news means discussing a poor prognosis					
Yes	136 (64%)	86 (69%)	1.94	1.03–3.64	0.039
No	75 (36%)	38 (31%)			
Breaking bad news is stressful					
Yes	78 (37%)	57 (46%)	2.92	1.49–5.73	0.001
No	133 (63%)	67 (54%)			
Discussing prognosis is talking about the success of treatment options					
Yes	75 (36%)	52 (42%)	2.12	1.05–4.28	0.036
No	136 (64%)	72 (58%)			
Self-evaluating as unskilled at patient-physician communication					
Yes	44 (22%)	31 (27%)	2.27	1.04–4.75	0.029
No	156 (78%)	85 (73%)			
Professional Role					
Resident	93 (46%)	60 (51%)	1.75	1.00–3.04	0.049
Consultant	109 (54%)	57 (49%)			
**Factors associated with low risk of burnout**					
Having a consistent plan for communication					
Yes	80 (38%)	40 (32%)	0.37	0.14–0.96	0.040
No	130 (62%)	84 (68%)			
Breaking bad news only considered as emotionally engaging					
Yes	158 (75%)	89 (72%)	0.56	0.31–0.98	0.042
No	53 (25%)	35 (28%)			
Addressing patients’ emotions with empathic responses					
Yes	137 (66%)	71 (58%)	0.39	0.18–0.85	0.017
No	72 (34%)	51 (42%)			
Discussing prognosis with the goal of promoting awareness of illness trajectory, therapeutic choices and to optimize patients’ adjustment					
Yes	119 (69%)	65 (63%)	0.44	0.24–0.82	0.010
No	53 (31%)	38 (37%)			
Sharing decisions with patients					
Yes	86 (41%)	42 (34%)	0.46	0.24–0.88	0.019
No	125 (59%)	82 (66%)			
Mastering communication skills by using textbooks and scientific literature					
Yes	13 (6%)	3 (2%)	0.18	0.01–0.68	0.011
No	198 (94%)	121 (98%)			
Self-evaluating communication skills as good or very good					
Yes	74 (35%)	30 (24%)	0.31	0.17–0.56	0.000
No	136 (75%)	93 (76%)			
Years from graduation					
6–16	60 (52%)	29 (23%^a^)	0.29	0.13–0.67	0.003
0–3	55 (48%)	37 (30%^a^)			

^a^The sum of these fractions is < 1 as the two compared covariates represent only a part of the whole responders cohort

Shown are only statistically significant associations. Percentages are calculated as fractions of responders to each item

*Abbreviations*: *OR* Odds Ratio, *CI* Confidence Interval

#### Multivariable analysis

The multivariable logistic regression model confirmed that physicians self-assessing BBN to be a stressful task for themselves (OR 3.01; 95% CI 1.26 to 7.19; *p* = 0.013) were associated with high levels of burnout; whereas a) physicians referring to plan in advance before communicating with patients (OR 0.43; 95% CI 0.21 to 0.89; *p* = 0.023), b) physicians reporting to have learnt CS from textbooks and scientific literature (OR 0.21; 0.05 to 0.93; *p* = 0.039) and c) physicians self-evaluating their ability to break bad news as good or very good (OR 0.32; 0.15 to 0.71; *p* = 0.005) were associated with low levels of burnout (Table 4). All other independent variables included in the multivariable model were not significantly associated to the presence of burnout. Goodness-of-fit of our multivariable model was good, as the c-statistic was equal to 0.78.

**Table 4:** Associations between communication patterns and burnout: multivariable analysis

Variables	OR	95% CI	***P*** value
**Factors associated with high risk of burnout**			
Breaking bad news is stressful	**3.01**	**1.26–7.19**	**0.013**
**Factors associated with low risk of burnout**			
Having a consistent plan for communication	0.43	**0.21–0.89**	**0.023**
Mastering communication skills by using textbooks and scientific literature	**0.21**	**0.05–0.93**	**0.039**
Self-evaluating communication skills as good or very good	**0.32**	**0.15–0.71**	**0.005**

Shown are only statistically significant associations

*Abbreviations*: *OR* Odds Ratio, *CI* Confidence Interval

## Discussion

This study collects descriptions and opinions of a sample of Italian hospital medical doctors on their own HC, including specific behaviours, thoughts, and feelings they might experience while getting ready for and performing difficult communication tasks. Moreover, it informs some of the factors and how they might relate to burnout metrics.

Results are consistent with those of previous surveys, mainly focused on the disclosure of the diagnosis, such as that from the American Society of Clinical Oncology [14, 16, 19, 22]. The majority of our respondents believed that BBN mainly equals discussing a poor prognosis, that discussing prognosis is the most difficult communication task, and that BBN is very emotionally engaging or stressful. Most clinicians admitted not using a consistent evidence-based framework for BBN encounter, not asking the patients the amount of information they want to receive, and checking for understanding only if they think this may be impaired. Fear is generally reported as the most frequently emotion raised in patients while discussing such topics. Respondents rated themselves good or at least fair in BBN and mostly reported acquiring CS empirically by observing colleagues. Of note, they reported very low rates of CS training both at medical school and beyond.

Also the frequency of burnout in our population is similar to that reported in US practising physicians, where nearly 60% of them experience the syndrome at some point in their career [2, 23, 24]. In Europe, similar rates were documented among French and Swiss physicians, 49 and 70%, respectively [25, 26]. Present data also confirm that younger medical doctors or residents have been reported to be exposed to an even higher risk [27].

The other important finding of our study is that, for the first time, it documents significant associations between some self-efficacy patterns regarding communication to patients and the risk of burnout. This study shows that physicians self-assessing BBN as a stressful task are exposed to a higher risk of burnout, up to three folds.

Previous researches have so far reported that BBN to patients have always been challenging for clinicians, either because many of them are concerned that honest information can damage patients’ hope or because they feel uncertain in managing patients’ emotion and estimating patients’ survival [28]. Indeed, by demonstrating the increase of several physiological indices (e.g. heart rate, blood pressure, skin conductance, cortisol levels, etc. …) during BBN encounters, other studies have empirically confirmed that physicians perceive BBN as a stressful task [9–11].

Our report supported by quantitative data suggests that these areas of self-efficacy, related to the distress, deriving from the uncertainty and the emotional burden, are linked to burnout.

Interestingly, clinicians for whom BBN means discussing a poor prognosis and who disclose prognosis only by talking about the success rate of therapies find themselves at a higher risk of burnout - although those were detected only in the single-item analysis. These findings suggest that the physicians’ uneasiness in discussing prognosis and the sole conscious positive estimate of treatment efficacy may have unintended consequences not only for patients, who may be led to seek life-sustaining therapies even in phases where active treatments will not be helpful, but also for physicians, who expose themselves to burnout, by risking losing patients’ trust when things get worse [29]. In the last few years, while new therapeutic technologies have progressively enabled patients to live longer with their disease than ever before, this has become even more complex [30].

Our data show that an evidence-based theoretical framework for the encounter may be protective of burnout in a statistically significant manner. This is even more important if we consider that the majority of our interviewed physicians admit not to plan a BBN encounter because of lack of time or because they consider this approach to be worthless. Previous qualitative studies found evidence that simple behavioral training has potential to positively affect physician-patient relationship and are felt beneficial by physician in terms of reducing BBN-related stress [31]. Our findings supported by quantitative data the effectiveness of this approach, and, together with the data that physicians who delay serious news discussions may experience high levels of burnout, further validate the importance of planning difficult communication tasks as a burnout prevention strategy. Furthermore, we found that physicians who are aware of communication skills by means of textbooks and scientific literature and those evaluating their ability to BBN at least good are exposed to low levels of burnout, in a statistically significant manner. Indeed, although understanding what patients want to know and delivering worrisome information may be stressful for clinicians, it has been reported that standard communication protocol may increase the confidence, the ability of physicians to disclose unfavourable medical information, eventually reducing the BBN related-stress, and may also increase patients’ rating of medical professionalism [32]. These findings, associated with the results of the single-item analysis, reporting low levels of burnout for physicians addressing patients’ emotions with empathy and fostering shared decision making, further support the relevance of acquiring, practising and improving basic CS as burnout prevention strategy [20].

Our study has several limitations. First, it was conducted on a sample of physicians who work in Modena, therefore the results we describe could not represent the entire national or international population. However, it should be recognised that a measurable rate of the interviewed physicians attended medical schools in different Italian regions, increasing at least in part the generalizability of the results. Second, the design of our study does not allow to establish an undoubted cause-effect association between the communication patterns and burnout metrics. Repeated monitoring of the same population over time would have consolidated the results. However, it has been recognised that the use of multiple assessments impairs similarly the reliability of the studies by increasing the likelihood of finding results. An ad-hoc survey was used and we acknowledge that objective measures of CST efficacy including the use of audio-recording of the medical encounters, for example, would provide more objective information about their communication habits. However, our data are consistent with the results of other surveys about communication and burnout rates in different countries and in different historical periods.

## Conclusions

In conclusion, our study suggests that physicians’ attitudes and practices about and during difficult communication tasks may influence their risk of burnout. These results support the relevance of embedding evidence-based communication skill training at all levels of professional medical development. Given the potential burnout impact for doctors it may be worth considering priority areas such as BBN and prognostication integrated with core CST to ensure they have mastered the foundation skills. Further studies on large number of physicians of different background and in different Countries are needed to confirm our results.

## Data Availability

The datasets used and/or analysed during the current study are available from the corresponding author on reasonable request.

## Abbreviations

BBN: Breaking bad news
PEE: Physical and emotional exhaustion
CD: Cynicism and depersonalization
PA: Personal accomplishment
HC: Healthcare communication
CS: Communication skills
MBI-HSS: Maslach Burnout Inventory - Human Services Survey
OR: Odds ratio
CI: Confidence interval
CST: Communication skill training
